# Health-related quality of life of children from low-income families: the new patterns study

**DOI:** 10.1186/s12889-023-17335-7

**Published:** 2023-12-06

**Authors:** Kristin Haraldstad, Eirik Abildsnes, Tormod Bøe, Kristine L. Vigsnes, Philip Wilson, Eirin Mølland

**Affiliations:** 1 Department of Health and Nursing science, Faculty of Health and Sport Science, University of Agder, Agder, Norway; 2 Department of Psychosocial Health, Faculty of Health and Sport Science, University of Agder, Agder, Norway; 3 Kristiansand Municipality, Agder, Norway; 4 Department of psychosocial science, Faculty of Psychology, University of Bergen, Bergen, Norway; 5 RKBU Vest, NORCE Norwegian Research Center, Bergen, Norway; 6 Institute of Applied Health Science, University of Aberdeen, Aberdeen, Scotland; 7 Centre for Research and Education in General Practice, University of Copenhagen, Copenhagen, Denmark; 8 Department of Economics and Finance, School of Business and Law, University of Agder, Agder, Norway; 9 NORCE, Norwegian Research Centre As, Bergen, Norway; 10 Department of Nutrition and Public Health, Faculty of Health and Sport Science, University of Agder, Agder, Norway

**Keywords:** Health-related quality of life, Well-being, Low-income families, Child poverty, Immigrant

## Abstract

**Background:**

Child poverty has been gradually rising, and about 12% of all Norwegian children are living in a state of relative poverty. This study was part of the New Patterns project, which recruits low-income families requiring long-term welfare services. Included families receive integrated welfare services, with the help of a family coordinator. The current study objectives were to explore the associations between HRQoL, demographic variables (age, gender, immigration status) and leisure activities in children and adolescents in low-income families.

**Methods:**

A cross-sectional survey was conducted among low-income families. Participating families had children (N = 214) aged 8–18 years.The family had a household income below 60% of the equivalized median population income for three consecutive years and needed long-term welfare services. HRQoL was measured using the KIDSCREEN-27 self-report instrument. Descriptive statistics, including means, standard deviations, and proportions, were calculated, and ordinary least squares regressions were performed, clustering standard errors at the family level.

**Results:**

Compared with boys, girls reported lower HRQoL on only one out of five dimensions, physical wellbeing. In the regression analysis we found statistically significant positive associations between migrant status and HRQoL on all five dimensions: physical wellbeing, psychological wellbeing, parents and autonomy, peers and social support, and school environment. In addition, age was associated with school environment, and age, gender and participation in leisure activities was associated with better physical wellbeing.

**Conclusions:**

Baseline results regarding HRQoL among children and adolescents in low-income families indicate that they have overall good HRQoL, though some participants had low HRQoL scores, especially on the physical and social support dimensions. Children with an immigrant background report higher HRQoL than do children without an immigrant background.

## Background

In recent years, gradually rising child poverty has become a cause for concern [1–3]. Compared with other countries, Norway has a low poverty rate. However, an increasing number of Norwegian children are growing up in poverty, as the country’s child poverty rate has tripled since 2001 [1]. About 12% of all Norwegian children now live in a state of relative poverty [3]. For some groups of immigrants, the risk of long-term poverty is more than 50% [1]. Particularly concerning are families because the increased poverty rate is more prevalent among them compared with the general population, and because children’s consequences may be significant [4]. In the recent report of health in inequalities in Norway, the needs of children of immigrants, and those in poverty is highlighted [5].

A longitudinal Norwegian study of intergenerational mobility demonstrated that children born into the lowest decile of the parental earnings rankings have fallen behind in several outcome dimensions [6]. This effect reflects employment, net household income, health (measured by disability program participation), family formation (for men), and mortality. Poverty affects the family as a whole and may have serious consequences for children, such as an increased risk of social marginalization and their own experience of poverty as adults [4]. Moreover, an extensive literature reports the negative health effects of poverty [7, 8]. For instance, a longitudinal study of adolescents in 34 countries indicated that those from low socioeconomic families are more affected by psychological and physical symptoms [9]. Poverty is also the most important social determinant of child health in high-income countries [10], and influences children’s health—largely indirectly, through adverse effects on their physical surroundings and psychosocial experiences [11, 12]. Accumulated poverty over time may result in adverse health outcomes [13, 14].

Leisure activities may be an important arena in children’s and adolescents’ lives. Adolescents of low socioeconomic status (SES) have been found to participate in fewer leisure activities compared to adolescents of high SES [15]. Participating in physical recreational activities has been shown to be related to better health and lower levels of depression and anxiety [16] Studying health-related quality of life (HRQoL) is important as it may provide a broad view of the impacts of poverty on child health [17–19]. HRQoL aims to assess various aspects of children’s health and well-being and has been described as “a multidimensional construct covering physical, emotional, social, and behavioral components of subjective well-being and functioning” [20]. The multidimensionality of the HRQoL concept provides researchers and practitioners with information about the multiple impacts of poverty and may serve as a framework for identifying and developing strategies to promote HRQoL [21]. The World Health Organization emphasizes well-being and HRQoL as public health goals and highlights child health and well-being as essential for healthier, more sustainable societies [17, 22, 23].

Relations between socioeconomic status (SES) and HRQoL are well established, with studies consistently demonstrating that low SES is related to low HRQoL [24–29]. A recent study from the Netherlands found that poverty at birth and/or cumulative poverty is associated with a significantly increased risk of lower HRQoL in children [10]. Different factors linking SES to HRQoL have been discussed, including differences in access to material and social resources and reactions to family stress [24]. Nevertheless, few studies have explored the dimensions of HRQoL among children living in poverty One reason for this is that compared with higher-income families, those living in poverty are less likely to participate in research and community programs [30].

The study described herein was part of the New Patterns project, which recruits low-income families requiring long-term welfare services. Participating families receive integrated services through a family coordinator across multiple sectors (culture, education, welfare, health, and social services), and volunteer organizations, supporting all family members for five years [31]. Studying the HRQoL of children growing up in poverty can provide important information for practice and policymakers and enhance the understanding of which aspects is relevant to health and HRQoL, especially for improving child well-being and reducing social inequity. Based on earlier research and literature, we focused on demographic variables, leisure activities in relation to HRQoL. The objectives of this study were to explore the associations between HRQoL, demographic variables (age, gender, immigration status) and leisure activities in children and adolescents in low-income families.

## Methods

### Study sample

The current cross-sectional study included 214 children (from 123 families) aged 8–18 years, mean age 12 years. The New Patterns project details, including recruitment and methodology, have been previously described [31]. Briefly, families were recruited through referrals from different service sectors within the municipalities. Eligible families had a household income below 60% of the equivalized median population income for three consecutive years and need for long-term welfare services. Participating families live in both urban and rural municipalities in southwestern Norway. Most of these participants (70%) enrolled in 2019 or 2020. Though project recruitment is ongoing, this study included only children who had enrolled by May 13, 2022.

### Data description

Families participating in the New Patterns project undergo a mapping process with a family coordinator, in close collaboration with all family members. Though mapping is repeated yearly, this study included only baseline results (i.e., within the first year of enrollment). Mapping details have also been reported by Mølland et al. (2021) [31]).

### Measurements

All background characteristics are measured at New Patterns project enrollment. The children answer the self-report questionnaires at home. The family coordinator is present and can answer questions if needed.

*Demographic variables*. The first part of the questionnaire included questions regarding demographic data, such as gender, age, parents education and immigration background and reason for immigration. Parental education is classified as the highest level of education completed by the mother and/or father at enrollment. Low education level means that neither parent (s) has completed more than mandatory education (10 years in Norway). Children categorized as having an immigrant background had either themselves immigrated to Norway or were born in Norway to a parent or parents who had immigrated there. Children born in Norway whose parents did not have an immigrant background were defined as having nonimmigrant background.

*HRQoL* was measured using the KIDSCREEN-27 self-report instrument [17], which was completed by each participating child. The KIDSCREEN-27 assess the subjective health and the psychological, mental, and social well-being (HRQoL) of children and adolescents between the ages of 8 and 18. The instrument assesses 5 dimensions : physical well-being (five items), psychological well-being (seven items), autonomy and parent relation (seven items), social support and peers (four items), and school environment (four items). Each item is rated on a five-point Likert scale referring to the past week, indicating either the intensity of an attitude or the frequency of a behavior or feeling. For example, the physical well-being dimension item “When thinking about the last week, have you been able to run well?” is rated using a scale from 1 (not at all) to 5 (extremely). Consistent with the KIDSCREEN manual, a scoring algorithm was used to convert the raw scores into T-scores [32]. Rasch scores are computed for each subscale and transformed into *t*-values normed to a mean (standard deviation [SD]) of 50 (10), which can be compared with international *t*-values [32]. If at least one item per dimension was left unanswered, the overall dimension score was identified as missing. Higher T-scores reflect better HRQoL. The Norwegian version has been shown to be valid and reliable [33].

*Participation in leisure activities* was measured with following item: Do you participate in organized free-time activities today? With *yes* or *no* as answer categories.

### Analysis

Data were analyzed using Stata 17 (Stata Corp. 2019, Stata Statistical Software: Release 16. College Station, TX, USA). Descriptive statistics, including means, SD, and fractions, were calculated. Age was grouped into two groups 8–11 and 12–18 in line with the recommendations from the KiDSCREEN group [32]. Differences in means were tested using standard *t* tests, assuming unequal variances. To explore relationships between HRQoL scores (dependent variables) and independent variables, age, gender demographic background and participation in leisure activities, ordinary least squares regressions were performed, clustering standard errors at the family level.

### Ethics

Informed consent was obtained from all participants, and informed consent was obtained from the parents of children younger than age 16 years. The study was approved by the Norwegian Regional Ethics Committee West (reference no. 249,507) and conducted according to recommendations from the Norwegian Data Protection Services (file no. 27,435).

## Results

Table 1 presents the 214 participants’ descriptive characteristics, including age, gender, and parental sociodemographics. Among the overall sample, girls were slightly overrepresented (54%), the mean age was 12 years, 68% had an immigrant background, 42% had parents who were married or cohabiting, and 58% had parents with low education. A large proportion of participants had parents who were not currently participating in the workforce (74%).

**Table 1:** Sample characteristics (*N* = 214)

	*N* (%)	Mean (SD)	Total observations
Age (years)		12.03 (2.55)	214
Girls	115 (54%)		214
Immigrant background	145 (68%)		214
Born in Norway	126 (59%)		214
Married/cohabitant parents	87 (42%)		209
Parents with low education level	122 (58%)		209
Parents who participated in the workforce in the last 12 months	75 (36%)		209
Parents currently participating in the workforce	56 (26%)		209

**Fig. 1:**
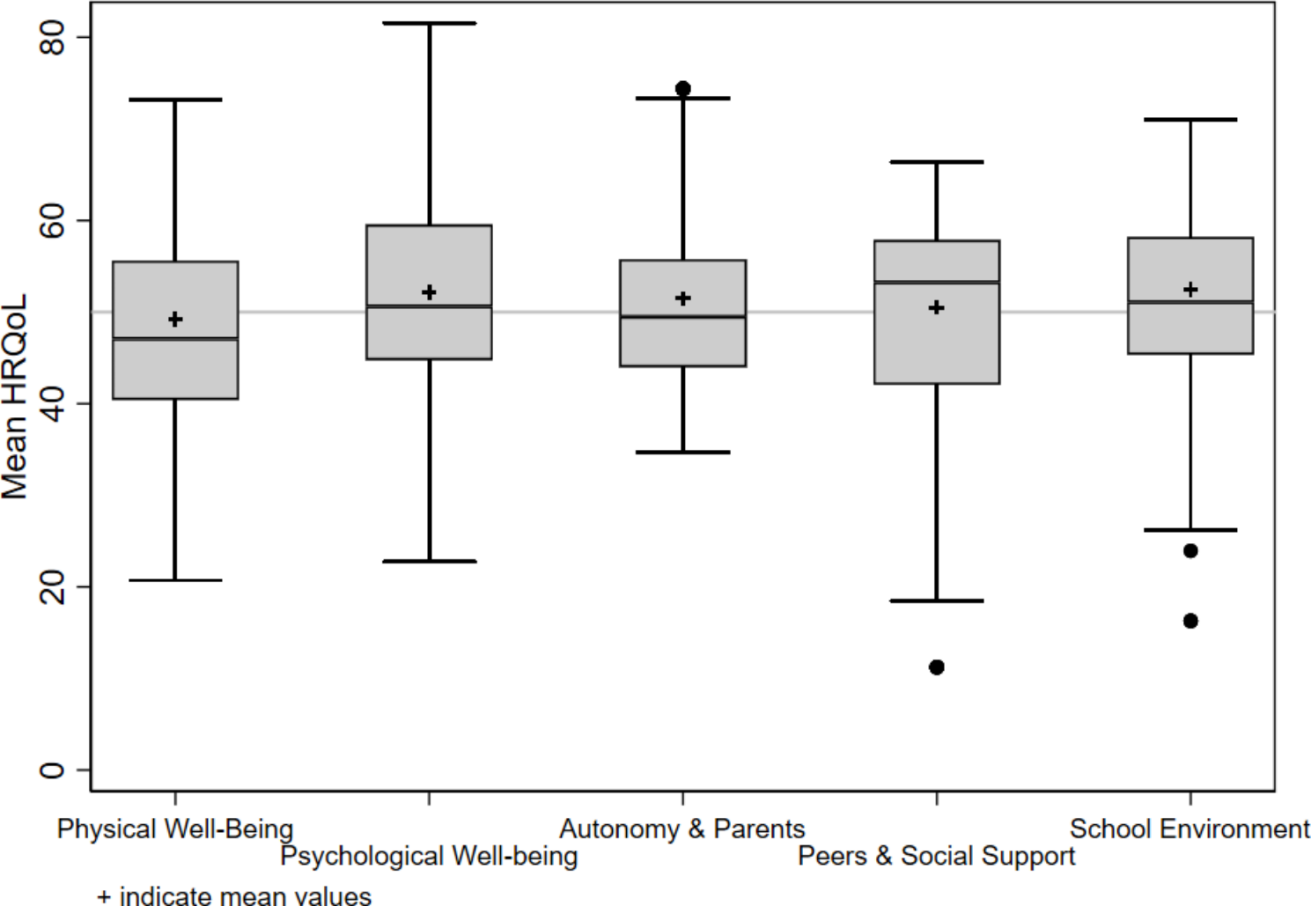
Mean self-reported health-related quality of life *Note:* Boxplot of health-related quality of life (HRQoL) dimensions. The boxes show the borders of the 25th and 75th percentiles for the *T*-scores of each HRQoL dimension. + indicates the mean *T*-score of each HRQoL dimension. Horizontal bold lines represent median *T*-scores for each HRQoL dimension. The gray line indicates the mean reference population *T*-score (50). Whiskers illustrate the lowest and highest *T*-scores, excluding outliers. Black dots outside whiskers represent outliers

Figure 1 shows a boxplot of the mean HRQoL scores for the five HRQoL domains for this sample, and European reference norms [32]. The plots indicate that the sample’s mean scores are close to European norms. The highest mean scores were for the domains of peer relationships and social support (mean 50.5, SD 12.3) and school environment (mean 52.4, SD 10.4), consistent with normative data. The lowest HRQoL score was for the physical well-being dimension. Although the mean scores were high for nearly all dimensions, there was a substantial range and variation within each dimension.

Table 2 presents the mean HRQoL scores and mean differences for age groups and gender. Age was grouped into categories: 8–11 years and 12–18 years. Children in the older group reported significantly lower HRQoL levels on the dimensions of physical well-being (mean difference 6.28; 95% confidence interval [CI] 3.13, 9.42) and school environment (mean difference 3.59; 95% CI 0.74, 6.44). The lowest HRQoL scores were reported for children in the older age group, on physical well-being (mean 46.50, SD 11.97).

**Table 2:** Mean self-reported health-related quality of life by age group and gender

	12–18 years	8–11 years	Mean difference	Cohen’s *d*	Girls	Boys	Mean difference	Cohen’s *d*
	Mean (SD)	Mean (SD)	Mean [95% CI]		Mean (SD)	Mean (SD)	Mean [95% CI]	
Physical	46.50	52.78	6.28***	0.53***	47.36	51.34	3.98*	0.33*
well-being	(11.97)	(11.37)	[3.13, 9.42]	[0.25, 0.82]	(12.09)	(11.82)	[0.77, 7.19]	[0.07, 0.59]
Psychological	51.83	52.62	0.79	0.073	51.11	53.37	2.25	0.21
well-being	(11.36)	(10.03)	[–2.18, 3.76]	[–0.18, 0.33]	(11.46)	(9.93)	[–0.70, 5.22]	[–0.062, 0.48]
Parents &	51.18	51.90	0.72	0.072	51.06	51.99	0.92	0.093
autonomy	(10.04)	(9.68)	[–1.95, 3.38]	[–0.191, 0.336]	(10.090)	(9.64)	[–1.72, 3.57]	[–0.19, 0.37]
Peers & social	50.19	50.92	0.72	0.058	50.38	50.65	0.27	0.022
support	(11.99)	(12.84)	[–2.65, 4.10]	[–0.237, 0.354]	(12.740)	(11.92)	[–3.032, 3.581]	[–0.25, 0.30]
School	50.89	54.48	3.59*	0.33*	52.19	52.71	0.52	0.047
environment	(11.59)	(9.64)	[0.74, 6.44]	[0.062, 0.60]	(11.20)	(10.62)	[–2.41, 3.44]	[–0.23, 0.32]
*N*	122	92	214	214	115	99	214	214

*Notes:* Mean differences in *T*-scores between boys and girls. 95% confidence intervals in brackets determined using a *t* test, assuming unequal variances.Cohen’s *d* with bootstrapped confidence intervals. * *p* < 0.05, ** *p* < 0.01, *** *p* < 0.001

Table 3 provides HRQoL information about children with and without an immigrant background and about participation in leisure activities. Children with an immigrant background reported higher HRQoL levels than children without an immigrant background on the dimensions: physical well-being (mean difference − 5.37; 95% CI − 8.58, − 2.25); psychological well-being (mean difference − 5.03; 95% CI − 8.06, − 2.00); parents and autonomy (mean difference − 3.44; 95% CI − 6.05, − 0.83); and school environment (mean difference − 5.19; 95% CI − 8.27, − 2.12).

**Table 3:** Mean self-reported health-related quality of life by immigrant status and participation in leisure activities

	immigrant	nonimmigrant	Mean difference	Cohen’s *d*	Participate in leisure activities	Do not participate in leisure activities	Mean difference	Cohen’s *d*
	Mean (SD)	Mean (SD)	Mean [95% CI]		Mean (SD)	Mean (SD)	Mean [95% CI]	
Physical	50.93	45.57	–5.37**	–0.45***	51.70	45.96	–5.74***	–0.48***
well-being	(12.42)	(10.60)	[–8.58, − 2.15]	[–0.72, − 0.18]	(11.90)	(11.63)	[–8.92, − 2.56]	[–0.77, − 0.19]
Psychological	53.78	48.75	–5.03**	–0.47**	53.737	50.212	–3.53*	–0.33*
well-being	(10.86)	(9.96)	[–8.06, − 2.00]	[–0.78, − 0.16]	(11.10)	(10.17)	[–6.47, − 0.57]	[–0.59, − 0.067]
Parents and	52.60	49.16	–3.44*	–0.35**	52.657	49.978	–2.68*	–0.27*
autonomy	(10.31)	(8.47)	[–6.05, − 0.83]	[–0.60, − 0.09]	(9.83)	(9.76)	[–5.32, − 0.03]	[–0.54, − 0.01]
Peers and social	51.60	48.19	–3.41	–0.27*	51.785	48.849	–2.93	–0.24
support	(12.46)	(11.83)	[–6.86, 0.04]	[–0.53, − 0.02]	(11.73)	(12.96)	[–6.29, 0.42]	[–0.51, 0.03]
School	54.11	48.92	–5.19**	–0.49***	53.231	51.407	–1.82	–0.17
environment	(10.61)	(10.77)	[–8.27, − 2.12]	[–0.76, − 0.21]	(10.46)	(11.45)	[–4.80, 1.15]	[–0.43, 0.09]
*N*	145	69	214	214	121	93	214	214

*Notes:* Mean differences in *T*-scores between children participating and not participating in leisure activities at the time of mapping. 95% confidence intervals in brackets determined using a *t* test, assuming unequal variances. Cohen’s *d* with bootstrapped confidence intervals. * *p* < 0.05, ** *p* < 0.01, *** *p* < 0.001

Children who participate in leisure activities reported higher levels of HRQoL on all dimensions, with significantly higher ratings on physical well-being (mean difference − 5.74; 95% CI − 8.92, − 2.56); psychological well-being (mean difference − 3.53; 95% CI − 6.47, − 0.57); parents and autonomy (mean difference − 2.68; 95% CI -5.32,-0.03).

Regression analysis was conducted to describe the relations between the independent variables and the dependent variables, the five HRQoL dimensions (Table 4). Models were adjusted for age, gender, participation in leisure activities, and immigrant background. Having an immigrant background was positively associated with all five HRQoL dimensions (i.e., physical well-being, psychological well-being, parents and autonomy, peers and social support, and school environment—indicating that children with immigrant backgrounds reported higher HRQoL than did children without an immigrant background. Age was negatively associated with the physical well-being and school environment dimensions.

**Table 4:** Regression results for health-related quality of life outcomes

	Physical well-being	Psychological well-being	Parents and autonomy	Peers and social support	School environment
	β (SE)	β(SE)	β (SE)	β(SE)	β (SE)
immigrant	5.14** (1.58)	5.00** (1.49)	3.46* (1.38)	3.48* (1.68)	5.17*** (1.49)
Age	–1.29*** (0.32)	–0.38 (0.31)	–0.10 (0.26)	–0.19 (0.32)	–1.05*** (0.27)
Male	3.32* (1.45)	1.66 (1.50)	0.39 (1.53)	–0.27 (1.62)	0.18 (1.50)
Participate in leisure activities	3.67* (1.84)	2.99 (1.69)	2.55 (1.58)	2.76 (1.81)	0.45 (1.59)
Constant	42.12*** (1.54)	46.38*** (1.58)	47.52*** (1.42)	46.71*** (1.92)	48.60*** (1.46)
Observations	214	201	214	214	214

*Notes* Each column is from a separate regression. *Immigrant* is a dummy variable indicating whether the child grew up/is growing up in an immigrant household. *Age* is centered. *Male* is a dummy variable indicating whether the child is male. Robust standard errors clustered at the family level. The first line in each row presents the estimated coefficients, whereas the second presents the standard errors (in parentheses)

* *p* < 0.05, ** *p* < 0.01, *** *p* < 0.001

## Discussion

These HRQoL results from children and adolescents in low-income families indicate that they have good overall HRQoL, with scores close to the average scores reported in previous national and European studies using the HRQoL instrument [17]. However, there were age and gender differences, as well as differences between children with and without immigrant background, and between those who participate and not participate in leisure activities.

A main study finding was the difference in HRQoL scores between participants with and without an immigrant background. The former reported significantly higher HRQoL than the latter on all five HRQoL dimensions physical and psychological well-being, parents and autonomy, peers and social support and school environment. Some earlier studies have indicated that a migration background can be a risk factor for health problems and low HRQoL [21, 33]. However, results have varied in the literature, with some studies indicating more similarities, and even higher mental health scores and HRQoL, among immigrant children compared with their nonimmigrant peers [34–37]. This variation has also been documented across ethnic groups [37]. In a German study, young children with an immigrant background reported higher HRQoL scores than did nonimmigrant children [38, 39]. Though it assessed both children and adults, one previous Norwegian study reported lower use of specialist mental service among immigrants compared with nonimmigrants [39]. Likewise, a recent study from Germany showed that children with low SES, low parental education, and a migrant status reported significantly lower HRQoL than did children without an immigrant background [40]. These inconsistencies suggest that such relations are highly complex.

The HRQoL differences between nonimmigrant and immigrant children from low-income families herein may be related to methodological challenges, including problems with survey completion because of language or cultural problems. However, differences may also be related to levels of family complexity [41]. Previous research has indicated that poverty can last across generations and that family characteristics can affect children over time, such as poor-quality parenting related to economic stress or parental health problems [8]. Children with a nonimmigrant background who grow up in poor families may therefore inherit less favorable family environments and experience more stigma related to their family situations [42, 43]. It should be noted that most families participating in our study that have a nonimmigrant background, also have at least one parent who is unable to work for different reason, such as health problems and disability. Another potential explanation for HRQoL differences may be that adolescents with an immigrant background experience less pressure from social referencing [44].

The HRQoL results in our study are in line with those of a recent study of adolescents from the general population in the same region of Norway [45]. We also found that younger children reported higher HRQoL than did older children, consistent with earlier findings that HRQoL is associated with age (i.e., older children report lower HRQoL than younger children) and gender (boys report higher HRQoL than girls) [21, 28, 46]. In general, our study participants had HRQoL scores close to the normative data on most dimensions. However, some participants had lower HRQoL than the European norms—especially on the physical and social support dimensions [32]. This is in line with a recent study from the Netherlands showing that children born into poverty have low physical HRQoL [47].

Herein, the lowest HRQoL scores were reported by the older children (12–18 years) on the physical well-being dimension, which was also more prevalent in girls than in boys. The physical well-being dimension explores children’s perceptions of their physical activity, health, and vitality [17] so that low scores indicate more impaired physical functioning. Children from poor families are more likely to develop a variety of health conditions. Unstable, low family income can lead to increased stress in the family and a lack of predictability [43]. In a systematic review, different mechanisms relating child poverty to health and well-being highlighted the importance of access to both material and social resources and child reactions to stress-inducing conditions [43]. The chronic stress of living in poverty can cause toxic stress [48], to which children may respond when they experience the accumulated burdens of family economic difficulties without adequate support [42, 49]. Children may express stress as complaints like headache and feeling in poor health [21, 50]. This stress experience, over time, may partly explain their low physical dimension score in this study.

Our results also show that children who participate in leisure activities report higher physical well-being dimension scores than those who do not. Participating in a variety of leisure activities can contribute positively to HRQoL in children by promoting physical health, emotional well-being, social development which is shown in previous studies [16, 51, 52], including a recent report on schoolchildren from a socioeconomically deprived area of England among whom there was a positive association between participation in leisure activities and HRQoL [51]. These findings may support the positive link between leisure activity engagement and HRQoL, though causality remains unclear.

### Strengths and limitations

The main study strength was its recruitment of children from low-income families who are less likely to participate in research and community programs, thereby describing an often-underrepresented population. We also recruited children with and without an immigrant background, using a validated HRQoL instrument. The findings should nevertheless be interpreted within the context of its limitations. The cross-sectional design disallows deducing causal relations; thus, longitudinal follow-up will be valuable.

## Conclusions

Baseline results regarding HRQoL among children and adolescents participating in an intervention targeting low-income families indicate that they have overall good HRQoL, though some participants had low HRQoL scores, especially on the physical and social support dimensions. Children with an immigrant background report higher HRQoL than do children without an immigrant background. HRQoL information in children from low-income families is vital for clinical practice and policy, especially toward improving child well-being.

## Data Availability

The datasets generated and analyzed for this study are not publicly available, due to regulation by the Norwegian Data Protection Authority, but are available from the corresponding author upon reasonable request.

## Abbreviations

CI: Confidence interval
SD: Standard deviation
HRQoL: Health-related Quality of Life
